# Bezold Abscess in a Case of Eosinophilic Otitis Media

**DOI:** 10.5811/cpcem.21313

**Published:** 2024-06-06

**Authors:** Satoshi Tsuruta, Takashi Fujiwara

**Affiliations:** Kurashiki Central Hospital, Department of Otolaryngology, Kurashiki city, Okayama, Japan

**Keywords:** *Mastoiditis*, *otitis media*, *abscess*, *eosinophilic otitis media*

## Abstract

**Case Presentation:**

A 57-year-old man with a history of bronchial asthma and eosinophilic sinusitis presented to the emergency department with an exacerbation of otitis media. His primary complaints were otopyorrhea, headache, and neck pain with redness. Contrast-enhanced computed tomography revealed a posterior neck abscess contiguous with the mastoid process. The patient underwent mastoidectomy and received antimicrobial therapy. Eosinophilic granulation tissue in the middle ear obstructed the middle ear aditus and directed the inflammatory process toward the mastoid tip.

**Discussion:**

Bezold abscess is a rare extracranial complication of acute mastoiditis. Therefore, clinicians should consider neck pain with redness as an important physical sign that suggests Bezold abscess in patients with otitis media.

CPC-EM Capsule What do we already know about this clinical entity?

*Patients with middle ear disease, particularly cholesteatoma, are at high risk of developing Bezold abscess.*

What is the major impact of the image(s)?

*This is the first reported case of Bezold abscess due to eosinophilic otitis media.*

How might this improve emergency medicine practice?

*
Emergency physicians should recognize that eosinophilic otitis media can cause the condition.*

## CASE PRESENTATION

**Image 1.**
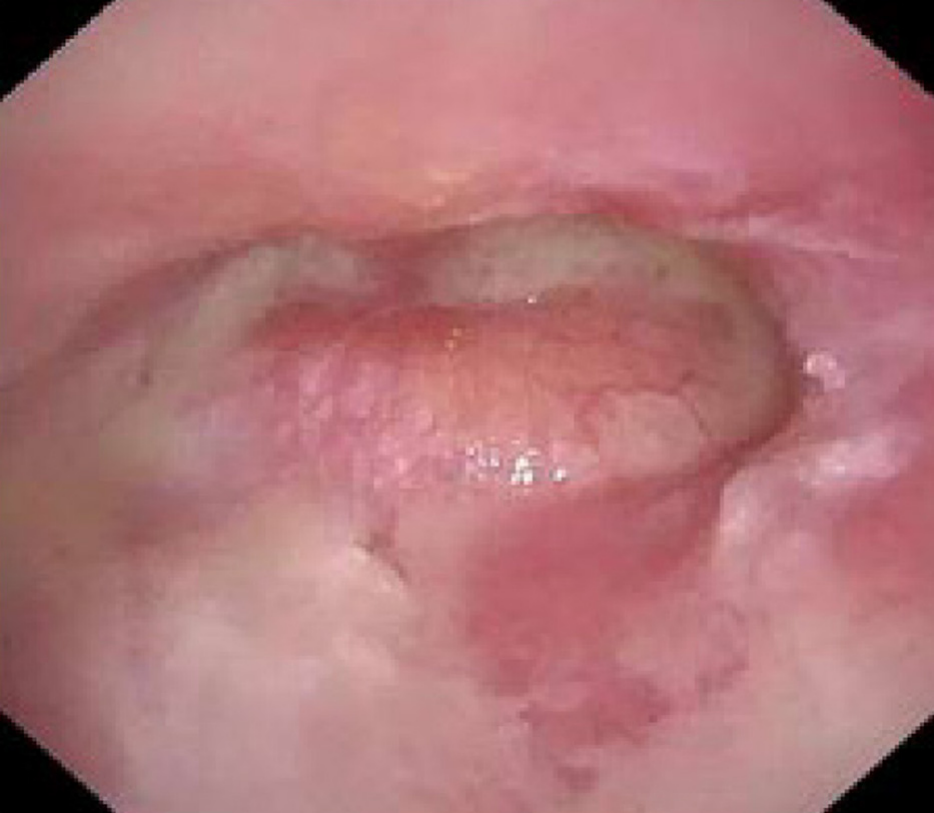
Otoscopic view of the middle ear demonstrating an erythematous, bulging tympanic membrane with purulent effusion.

**Image 2.**
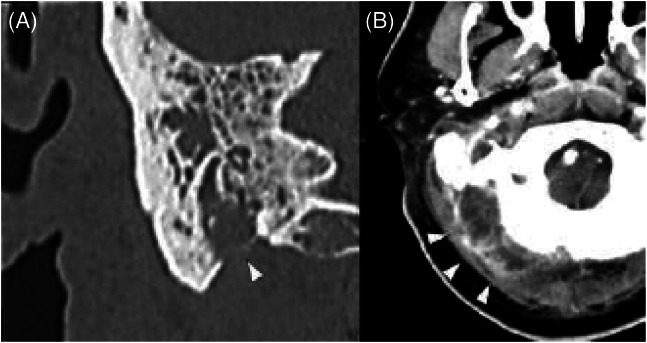
(A) Computed tomography (CT) of the mastoid with intravenous (IV) contrast showing mastoid process perforation (arrowhead); (B) CT of the neck with IV contrast demonstrating a posterior neck abscess (arrowheads).

A 57-year-old man with a history of bronchial asthma and eosinophilic sinusitis presented to the emergency department with an exacerbation of otitis media. He had previously received treatment with tympanostomy and antimicrobial therapy for acute otitis media of his right ear, but his condition did not improve. He experienced otopyorrhea (Image 1), worsening pain in his right ear and neck with redness, headache, and impaired consciousness. Contrast-enhanced computed tomography revealed bilateral otitis media, thrombosis from the right internal jugular vein to the sigmoid sinus, and a posterior neck abscess contiguous with the mastoid process on the right side (Image 2).

**Image 3.**
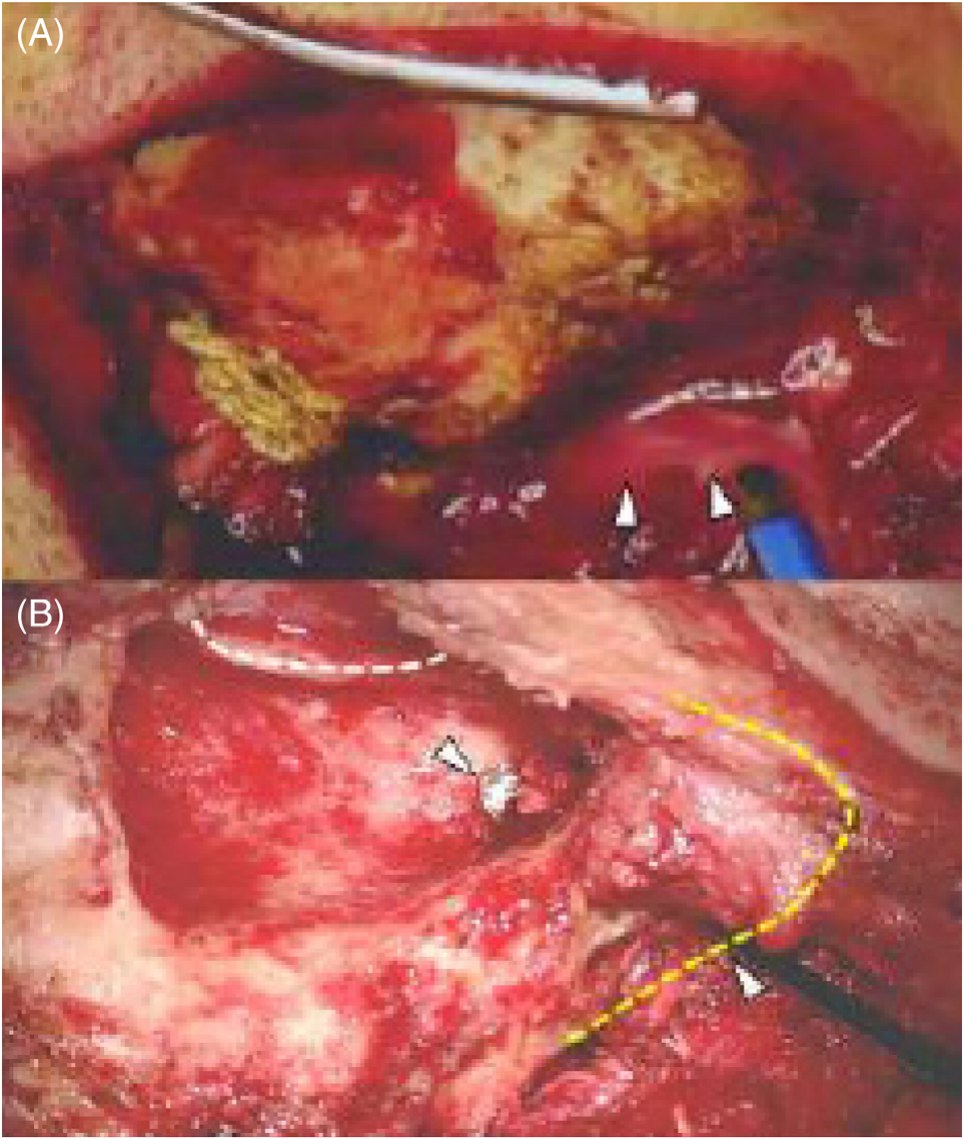
Intraoperative images during mastoidectomy. (A) Before mastoidectomy: Drainage of pus from the mastoid process was observed (arrowheads). (B) After mastoidectomy: The dashed white line represents the posterior wall of the ear canal, whereas the dashed yellow line indicates the mastoid tip. A path between the mastoid antrum and neck is visible (arrowhead), through which surgical instruments could be inserted from the neck to the mastoid antrum.

The patient underwent mastoidectomy and was treated with antimicrobial and antithrombotic therapies. During surgery, it was found that the right middle ear was filled with eosinophilic granulation tissue, with the formation of a path between the mastoid part of the temporal bone and the posterior neck (Image 3). Intraoperative intratympanic steroid administration eliminated the granulation tissue a few days after the surgery. The patient was treated with antimicrobial therapy for six weeks. He was discharged with higher functional impairment and gait disturbance. However, he continued his rehabilitation and could drive two months after discharge. Furthermore, the patient successfully reintegrated into society and was found to be in good health one year later.

## DISCUSSION

Bezold abscess, a rare extracranial complication of acute mastoiditis, occurs when an infection erodes through the lateral mastoid cortex medially toward the neck. Although the incidence of Bezold abscess has decreased with the development of antimicrobial agents and improved nutrition, individuals with middle ear disease, particularly cholesteatoma, remain at a higher risk of developing this condition. The presence of middle ear disease (eg, cholesteatoma) can obstruct the middle ear aditus and direct the inflammatory process toward the mastoid tip. In the present case, Bezold abscess occurred despite the administration of appropriate antimicrobial agents and tympanostomy.

The patient had a history of bronchial asthma and eosinophilic sinusitis, leading to the diagnosis of eosinophilic otitis media. Acute otitis media exacerbates the development of eosinophilic granulation tissue in the middle ear, which can obstruct the middle ear aditus, leading to neck abscess formation. To our knowledge, this is the first case of Bezold abscess due to eosinophilic otitis media. Thus, emergency physicians should recognize that eosinophilic otitis media can cause Bezold abscess.

## References

[1] Alkhaldi AS Alwabili M Albilasi T et al . Bezold’s abscess: a case report and review of cases over 20 years. Cureus. 2022;14(1):e21533. 35223308 10.7759/cureus.21533 PMC8863901

[2] Uchida Y Ueda H Nakashima T . Bezold’s abscess arising with recurrent cholesteatoma 20 years after the first surgery: with a review of the 18 cases published in Japan since 1960. Auris Nasus Larynx. 2002;29(4):375–8. 12393045 10.1016/s0385-8146(02)00057-3

